# Author Correction: Biallelic variants in the noncoding RNA gene *RNU4-2* cause a recessive neurodevelopmental syndrome with distinct white matter changes

**DOI:** 10.1038/s41588-026-02636-5

**Published:** 2026-05-18

**Authors:** Rocio Rius, Alexander J. M. Blakes, Yuyang Chen, Joachim De Jonghe, François Lecoquierre, Ruebena Dawes, Benjamin Cogne, Hyung Chul Kim, Javeria R. Alvi, Florence Amblard, Morad Ansari, Annabelle Arlt, Christina Austin-Tse, Sarah Baer, Meena Balasubramanian, Elsa V. Balton, Giulia Barcia, Ana Beleza-Meireles, Jonathan A. Bernstein, Jasmin Beygo, Pierre Blanc, Nuria C. Bramswig, Frederik Braun, Daniel Buchzik, Daniel G. Calame, Jamie Campbell, Charles Coutton, Chloe A. Cunningham, Nitsuh Dargie, Christel Depienne, Katrina M. Dipple, Anne Dieux, Abhijit Dixit, Lauren Dreyer, Haowei Du, Salima El Chehadeh, Michael Field, Lisa J. Ewans, Vanessa Geiger, Richard A. Gibbs, Ian Glass, Olivier Grunewald, Paul Gueguen, Tobias B. Haack, Hamza Hadj Abdallah, Radu Harbuz, Ingo Helbig, Judit Horvath, Alexander Hustinx, Bertrand Isidor, Marie-Line Jacquemont, Fraser Jamie, Médéric Jeanne, Riley Kessler, Hannah Klinkhammer, G. Christoph Korenke, Urania Kotzaeridou, Peter Krawitz, Steven Laurie, Richard J. Leventer, Rebecca J. Levy, James R. Lupski, Pierre Marijon, Kaitlin E. McGinnis, Rodrigo Mendez, Olfa Messaoud, Caroline Nava, Mevyn Nizard, Anne O’Donnell-Luria, Melanie C. O’Leary, Simone Olivieri, Amitav Parida, Davut Pehlivan, Anna Jenne Prentice, Jennifer E. Posey, Chloe M. Reuter, Véronique Satre, Caroline Schluth-Bolard, Thomas Smol, Tipu Sultan, John Taylor, Christel Thauvin-Robinet, Julien Thevenon, Eloise Uebergang, Sandra Ueberberg, Catherine Vincent-Delorme, Evangeline Wassmer, Emma Westwood, Matthew T. Wheeler, Elif Yilmaz Gulec, Adeline Vanderver, Arastoo Vossough, Stephan J. Sanders, Siddharth Banka, Gregory M. Findlay, Daniel G. MacArthur, Cas Simons, Nicola Whiffin

**Affiliations:** 1 Centre for Population Genomics, Garvan Institute of Medical Research, UNSW Sydney, Sydney, New South Wales Australia; 2 Centre for Population Genomics, Murdoch Children’s Research Institute, Melbourne, Victoria Australia; 3 Manchester Centre for Genomic Medicine, Division of Evolution and Genomic Sciences, School of Biological Sciences, Faculty of Biology, Medicine and Health, University of Manchester, Manchester, UK; 4 Manchester Centre for Genomic Medicine, St Mary’s Hospital, Manchester University NHS Foundation Trust, Health Innovation Manchester, Manchester, UK; 5 Big Data Institute, University of Oxford, Oxford, UK; 6 Centre for Human Genetics, University of Oxford, Oxford, UK; 7 The Genome Function Laboratory, The Francis Crick Institute, London, UK; 8 Univ Rouen Normandie, Inserm U1245 and CHU Rouen, Department of Genetics and Reference Center for Developmental Abnormalities, Rouen, France; 9 Nantes Université, CHU de Nantes, CNRS, INSERM, l’institut du thorax, Nantes, France; 10 Nantes Université, CHU de Nantes, CNRS, INSERM, Génétique médicale, Nantes, France; 11 Department of Pediatric Neurology, University of Child Health Sciences, The Children’s Hospital, Lahore, Pakistan; 12 Service de Génétique, Génomique et Procréation, CHU Grenoble Alpes, Grenoble, France; 13 GCS AURAGEN, Lyon, France; 14 Université Grenoble Alpes, INSERM U 1209, CNRS UMR 5309, Institut for Advanced Biosciences, Grenoble, France; 15 South East Scotland Clinical Genetics Service, NHS lothian, Edinburgh, UK; 16 Institute for Genomic Statistics and Bioinformatics, Medical Faculty, University of Bonn, Bonn, Germany; 17 Center for Mendelian Genomics, Program in Medical and Population Genetics, Broad Institute of MIT and Harvard, Cambridge, MA USA; 18 Center for Genomic Medicine, Massachusetts General Hospital, Boston, MA USA; 19 Department of Pathology, Harvard Medical School, Boston, MA USA; 20 Department of Neuropediatrics, ERN EpiCare, French Centre de référence des Épilepsies Rares (CréER), Hôpitaux Universitaires de Strasbourg, Strasbourg, France; 21 Institute for Genetics and Molecular and Cellular Biology (IGBMC), University of Strasbourg, CNRS UMR7104, Illkirch, France; 22 Division of Clinical Medicine, School of Medicine and Population Health, University of Sheffield, Sheffield, UK; 23 Sheffield Clinical Genomics Service, Sheffield Children’s NHS Foundation Trust, Sheffield, UK; 24 Department of Medicine, University of Washington School of Medicine, Seattle, WA USA; 25 Genomic Medecine of Rare Disease, Necker Hospital, Paris, France; 26 Imagine Institute, Paris, France; 27 Clinical Genetics Department, Guy’s and St Thomas’ NHS Foundation Trust, London, UK; 28 Department of Pediatrics, Stanford University School of Medicine, Stanford, CA USA; 29 Institute of Human Genetics, University Hospital Essen, University Duisburg-Essen, Essen, Germany; 30 Laboratoire SeqOIA, Paris, France; 31 Department of Medical Genetics, Centre of Medical Genetics, University and University Hospital Münster, Münster, Germany; 32 Department of Neuropediatrics, Diak Klinikum Landkreis Schwäbisch Hall, Schwäbisch Hall, Germany; 33 Section of Pediatric Neurology, Department of Pediatrics, Baylor College of Medicine, Houston, TX USA; 34 Texas Children’s Hospital, Houston, TX USA; 35 Victorian Clinical Genetics Services, Murdoch Children’s Research Institute, Melbourne, Victoria Australia; 36 Department of Paediatrics, University of Melbourne, Melbourne, Victoria Australia; 37 Department of Pediatrics, University of Washington, Seattle, WA USA; 38 Brotman Baty Institute for Precision Medicine, Seattle, WA USA; 39 CHU Lille, ULR7364 – RADEME – Maladies Rares du Développement Embryonnaire, Lille, France; 40 Clinique de Génétique, Hôpital Jeanne de Flandre, CHU de Lille, Lille, France; 41 Clinical Genetics, Nottingham University Hospitals, Nottingham, UK; 42 Genetic Health WA, Perth, Western Australia Australia; 43 Department of Molecular and Human Genetics, Baylor College of Medicine, Houston, TX USA; 44 Service de Génétique Médicale, Institut de Génétique Médicale D’Alsace, Hôpitaux Universitaires de Strasbourg, Strasbourg, France; 45 Laboratoire de Génétique Médicale, Institut de Génétique Médicale d’Alsace, INSERM UMRS_1112, CRBS, Université de Strasbourg, Strasbourg, France; 46 Genetics of Learning Disability Service, Hunter Genetics, Waratah, Western Australia Australia; 47 Centre for Clinical Genetics, Sydney Children’s Hospitals Network, Sydney, New South Wales Australia; 48 Genomics and Inherited Diseases Program, Garvan Institute of Medical Research, Darlinghurst, New South Wales Australia; 49 Discipline of Paediatrics and Child Health, School of Clinical Medicine, Faculty of Medicine and Health, University of New South Wales, Sydney, New South Wales Australia; 50 Genetikum, MVZ genetikum GmbH, Neu-Ulm, Germany; 51 Human Genome Sequencing Center, Baylor College of Medicine, Houston, TX USA; 52 U1172-LilNCog-Lille Neuroscience & Cognition, CHU de Lille, Lille, France; 53 Laboratoire de Genopathies, CHU Lille, Lille, France; 54 Service de Génétique, CHRU de Tours, Tours, France; 55 Université de Tours, INSERM, Imaging Brain & Neuropsychiatry iBraiN, Tours, France; 56 Institute of Medical Genetics and Applied Genomics, Eberhard Karls University, Tübingen, Germany; 57 The Epilepsy NeuroGenetics Initiative (ENGIN), Children’s Hospital of Philadelphia, Philadelphia, PA USA; 58 Epilepsy and Neurodevelopmental Disorders Center (ENDD), Children’s Hospital of Philadelphia, Philadelphia, PA USA; 59 Division of Neurology, Department of Pediatrics, Children’s Hospital of Philadelphia, Philadelphia, PA USA; 60 Department of Neurology, Perelman School of Medicine, University of Pennsylvania, Philadelphia, PA USA; 61 Service de Génétique Médicale, Institut de Génétique Médicale D’Alsace, Centre Hospitalier Universitaire de Nantes, Nantes, France; 62 Centre de Référence Maladies Rares “Anomalies du Développement et Syndromes Malformatifs”, FHU Genomeds, CHRU de Tours, Tours, France; 63 Rare Disease Institute, Division of Genetics and Metabolism and Center for Genetic Medicine Research, Children’s National Hospital, Washington, DC USA; 64 PRISME division for congenital and Developmental Disorders, Department of Genetics, Hôpital de l’Estran, Avranches, France; 65 Institute for Medical Biometry and Statistics, Marburg University, Marburg, Germany; 66 Department of Neuropediatrics, University Children’s Hospital, Klinikum Oldenburg, Oldenburg, Germany; 67 Department of Pediatrics I, Division of Pediatric Neurology and Metabolic Medicine, Medical Faculty of Heidelberg, Heidelberg, Germany; 68 Centro Nacional de Análisis Genómico (CNAG), Baldiri Reixac 4, Barcelona, Spain; 69 Universitat de Barcelona (UB), Barcelona, Spain; 70 Murdoch Children’s Research Institute, Melbourne, Victoria Australia; 71 Royal Children’s Hospital, Melbourne, Victoria Australia; 72 Division of Child Neurology, Department of Neurology and Neurological Sciences, Stanford University, Stanford, CA USA; 73 Department of Pediatrics, Baylor College of Medicine, Houston, TX USA; 74 Cardiovascular Medicine, Stanford University, Stanford, CA USA; 75 Division of Genetics and Genomics, Boston Children’s Hospital, Boston, MA USA; 76 Harvard Medical School, Boston, MA USA; 77 Sorbonne Université, Institut du Cerveau - Paris Brain Institute - ICM, Inserm, CNRS, APHP, Département de Génétique, Hôpital de la Pitié Salpêtrière, Paris, France; 78 Paris Cité University, Paris, France; 79 Department of Pediatrics, Harvard Medical School, Boston, MA USA; 80 Department of Paediatric Neurology, Birmingham Women’s and Children’s Hospital Foundation Trust, Birmingham, UK; 81 Laboratoire de Diagnostic Génétique, Institut de Génétique Médicale d’Alsace, INSERM UMRS_1112, Université de Strasbourg, Hôpitaux Universitaires de Strasbourg, Strasbourg, France; 82 Department of Radiology, NHS lothian, Edinburgh, UK; 83 Université Bourgogne Europe - CHU Dijon Bourgogne - Inserm U1231 CTM GAD, Centre de Référence des maladies neurogénétiques, Laboratoire de Génomique Médicale, Dijon, France; 84 Consultation de génétique, CH Arras, Arras, France; 85 Birmingham Children’s Hospital, Birmingham, UK; 86 Institute of Health and Neurodevelopment, Aston University, Birmingham, UK; 87 NHS Education for Scotland, NHS Scotland, Edinburgh, UK; 88 Department of Medical Genetics, Istanbul Medeniyet University Medical School, Istanbul, Turkey; 89 Medical Genetics Clinic, Istanbul Goztepe Prof Dr Suleyman Yalcin City Hospital, Istanbul, Turkey; 90 Department of Radiology, Children’s Hospital of Philadelphia, Philadelphia, PA USA; 91 Institute of Developmental and Regenerative Medicine, Department of Paediatrics, University of Oxford, Oxford, UK; 92 Department of Psychiatry and Behavioral Sciences, UCSF Weill Institute for Neurosciences, University of California San Francisco, San Francisco, CA USA

**Keywords:** Genomics, Medical genetics, Genetic testing

Correction to: *Nature Genetics* 10.1038/s41588-026-02554-6, published online 8 April 2026.

In the version of this article initially published, the last name of Christel Thauvin-Robinet was misspelled (Thauvin-Robinetvin) and is now amended in the HTML and PDF versions of the article.

