# Supplementary material for: Biallelic variants in the noncoding RNA gene RNU4-2 cause a recessive neurodevelopmental syndrome with distinct white matter changes
Source: Nat Genet. 2026 Apr 8;58(4):761–73. doi: 10.1038/s41588-026-02554-6 (PMC13083247; doi:10.1038/s41588-026-02554-6)
Supplement: Supplementary file 1 — Reporting Summary [file 41588_2026_2554_MOESM1_ESM.pdf]

nature portfolio

Corresponding author(s): Whiffin, Simons

Last updated by author(s): Feb 19, 2026

Reporting Summary

Nature Portfolio wishes to improve the reproducibility of the work that we publish. This form provides structure for consistency and transparency in reporting. For further information on Nature Portfolio policies, see our Editorial Policies and the Editorial Policy Checklist.

Statistics

For all statistical analyses, confirm that the following items are present in the figure legend, table legend, main text, or Methods section.

n/a	Confirmed	
☐	☒	The exact sample size (*n*) for each experimental group/condition, given as a discrete number and unit of measurement
☒	☐	A statement on whether measurements were taken from distinct samples or whether the same sample was measured repeatedly
☐	☒	The statistical test(s) used AND whether they are one- or two-sided<br>*Only common tests should be described solely by name; describe more complex techniques in the Methods section.*
☐	☒	A description of all covariates tested
☐	☒	A description of any assumptions or corrections, such as tests of normality and adjustment for multiple comparisons
☐	☒	A full description of the statistical parameters including central tendency (e.g. means) or other basic estimates (e.g. regression coefficient) AND variation (e.g. standard deviation) or associated estimates of uncertainty (e.g. confidence intervals)
☐	☒	For null hypothesis testing, the test statistic (e.g. F, t, r) with confidence intervals, effect sizes, degrees of freedom and P value noted<br>*Give P values as exact values whenever suitable.*
☒	☐	For Bayesian analysis, information on the choice of priors and Markov chain Monte Carlo settings
☒	☐	For hierarchical and complex designs, identification of the appropriate level for tests and full reporting of outcomes
☐	☒	Estimates of effect sizes (e.g. Cohen's *d*, Pearson's *r*), indicating how they were calculated

Our web collection on statistics for biologists contains articles on many of the points above.

Software and code

Policy information about availability of computer code

Data collection	Clinical data were collated using Google sheets and Microsoft Excel spreadsheets.
Data analysis	This study predominantly used available software packages: bedtools (v2.31.0), bcftools (v1.16), and samtools (v1.9). R (v4.1.1) was used via RStudio with plots generated using ggplot (v3.5.2) and related packages. Code generated for analyses within this manuscript is stored securely in the National Genomics Research Library and available to be shared within this environment upon request.

For manuscripts utilizing custom algorithms or software that are central to the research but not yet described in published literature, software must be made available to editors and reviewers. We strongly encourage code deposition in a community repository (e.g. GitHub). See the Nature Portfolio guidelines for submitting code & software for further information.

Data

Policy information about availability of data

All manuscripts must include a data availability statement. This statement should provide the following information, where applicable:

- Accession codes, unique identifiers, or web links for publicly available datasets
- A description of any restrictions on data availability
- For clinical datasets or third party data, please ensure that the statement adheres to our policy

Data from the National Genomic Research Library (NGRL) used in this research are available within the secure Genomics England Research Environment. Access to NGRL data is restricted to adhere to consent requirements and protect participant privacy. Access to NGRL data is provided to approved researchers who are

members of the Genomics England Research Network, subject to institutional access agreements and research project approval under participant-led governance. For more information on data access, visit: https://www.genomicsengland.co.uk/research

Genomic and phenotypic data from the GREGoR consortium (including the RGP cohort) and the UDN are available through the dbGaP accession numbers phs003047.v1.p1 and phs001232.v5.p2, respectively, with at least annual data releases. Access is managed by a data access committee designated by dbGaP and is based on intended use of the requester and allowed use of the data submitter as defined by consent codes.

Data access to individual genome data from the PFMG2025 with other researchers is submitted to current data protection and regulations in France and is only possible through the Collecteur Analyseur de Données (CAD). More information on data access and the CAD structure can be obtained on the PFMG2025 website (https://pfmg2025.fr/le-plan/collecteur-analyseur-de-donnees-cad/).

UK Biobank data are available in the Research Analysis Platform (UKB-RAP) for use by eligible researchers under approved access. This research was conducted under application number 81050.

RNA-sequencing data in Figure 6A is available in EGA (accession: EGAS50000000889). Data from Figures B-D is available within the NGRL (see access details above).

Variant curations have been deposited in ClinVar under submission SUB16002064 (accessions SCV007494689 - SCV007494723).

All other data are available within the manuscript and supplementary tables.

Research involving human participants, their data, or biological material

Policy information about studies with human participants or human data. See also policy information about sex, gender (identity/presentation), and sexual orientation and race, ethnicity and racism.

Reporting on sex and gender	We use the term sex to describe individuals.
Reporting on race, ethnicity, or other socially relevant groupings	We record reported ancestry of a subset of participants with detailed clinical information. These data are not used in any analyses.
Population characteristics	31 individuals with biallelic RNU4-2 variants had detailed phenotype characterisation. 21 of these individuals were male, with an average age of 10.
Recruitment	Participants were recruited to large genomic sequencing cohorts (e.g. Genomics England) based on clinical presentation. There could be biases from accessibility to recruitment centres.
Ethics oversight	Informed consent was obtained for all patients included in this study from their parent(s) or legal guardian, with the study approved by the local regulatory authority. A specific consent form was obtained from the families who consented to the publication of photographs. The 100,000 Genomes Project Protocol has ethical approval from the HRA Committee East of England Cambridge South (REC Ref 14/EE/1112). This study was registered with Genomics England under Research Registry Projects 354. Health related research in UK Biobank was approved by the Research Ethics Committee (REC) under reference 16/NW/0274 with this research conducted under application number 81050.

Note that full information on the approval of the study protocol must also be provided in the manuscript.

Field-specific reporting

Please select the one below that is the best fit for your research. If you are not sure, read the appropriate sections before making your selection.

☒ Life sciences ☐ Behavioural & social sciences ☐ Ecological, evolutionary & environmental sciences

For a reference copy of the document with all sections, see nature.com/documents/nr-reporting-summary-flat.pdf

Life sciences study design

All studies must disclose on these points even when the disclosure is negative.

Sample size	Sample size was not predetermined, but was based on prior recruitment to genome sequencing cohorts. All undiagnosed individuals with neurodevelopmental disorders were included.
Data exclusions	No data were excluded from the analyses.
Replication	Replication was achieved by identifying individuals across multiple rare disease cohorts. No other form of replication is relevant to this study as no experiments were performed.
Randomization	This is an observational study so randomisation is not relevant.
Blinding	This is an observational study so blinding is not relevant.

Reporting for specific materials, systems and methods

We require information from authors about some types of materials, experimental systems and methods used in many studies. Here, indicate whether each material, system or method listed is relevant to your study. If you are not sure if a list item applies to your research, read the appropriate section before selecting a response.

Materials & experimental systems

n/a	Involved in the study
☒	☐ Antibodies
☒	☐ Eukaryotic cell lines
☒	☐ Palaeontology and archaeology
☒	☐ Animals and other organisms
☒	☐ Clinical data
☒	☐ Dual use research of concern
☒	☐ Plants

Methods

n/a	Involved in the study
☒	☐ ChIP-seq
☒	☐ Flow cytometry
☒	☐ MRI-based neuroimaging

Plants

Seed stocks	NA
Novel plant genotypes	NA
Authentication	NA
